# Flail arm–like syndrome associated with HIV-1 infection

**DOI:** 10.4103/0972-2327.53084

**Published:** 2009

**Authors:** A. Nalini, Anita Desai, Simendra Kumar Mahato

**Affiliations:** Department of Neurology, National Institute of Mental Health and Neurosciences, Bangalore, India; 1 Department of Neurovirology, National Institute of Mental Health and Neurosciences, Bangalore, India; 2 Department of Neuroradiology, National Institute of Mental Health and Neurosciences, Bangalore, India

**Keywords:** Flail arm syndrome, HIV-1, amyotrophic lateral sclerosis, highly active antiretroviral therapy, HIV-1 clade C

## Abstract

During the last 20 years at least 23 cases of motor neuron disease have been reported in HIV-1 seropositive patients. In this report we describe the clinical picture of a young man with HIV-1 clade C infection and flail arm-like syndrome, who we were able to follow-up for a long period. We investigated and prospectively monitored a 34-year-old man with features of flail arm syndrome, who developed the weakness and wasting 1 year after being diagnosed with HIV-1 infection after a routine blood test. He presented in 2003 with progressive, symmetrical wasting and weakness of the proximal muscles of the upper limb of 2 years' duration. He had severe wasting and weakness of the shoulder and arm muscles. There were no pyramidal signs. He has been on HAART for the last 4 years and the weakness or wasting has not worsened. At the last follow-up in July 2007, the patient had the same neurological deficit and no other symptoms or signs of HIV-1 infection. MRI of the spinal cord in 2007 showed characteristic T2 hyperintense signals in the central part of the spinal cord, corresponding to the central gray matter. Thus, our patient had HIV-1 clade C infection associated with a ‘flail arm–like syndrome.’ The causal relationship between HIV-1 infection and amyotrophic lateral sclerosis (ALS)-like syndrome is still uncertain. The syndrome usually manifests as a lower motor neuron syndrome, as was seen in our young patient. It is known that treatment with antiretroviral therapy (ART) stabilizes/improves the condition. In our patient the weakness and atrophy remained stable over a period of 3.5 years after commencing HAART regimen.

## Introduction

HIV-associated motor neuron disease (MND) was first reported in 1985, while AIDS itself was described initially in 1981.[1] There are several reports of a MND- like syndrome in HIV-1 infection. Although HIV-1–associated MND may be indistinguishable from classical amyotrophic lateral sclerosis (ALS),[2–4] more frequently it is characterized by a variably progressive lower motor neuron disorder affecting the limb or bulbar muscles.[4–6] The flail arm syndrome, a variant of MND, is characterized by a relatively symmetric involvement of the proximal muscles of both arms, which progresses to severe wasting and functional disability; there is, however, little or no weakness of the leg or bulbar muscles at presentation.[7] The median survival of these patients is reported to be 57 months.[8] Another term used for a similar syndrome is amyotrophic brachial diplegia.[9] Here we report a case of a young man with a flail arm–like syndrome associated with HIV-1 clade C infection.

## Case Report

**Figure 1:**
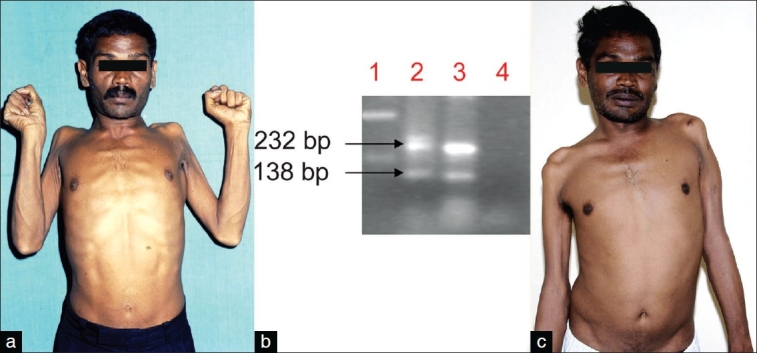
(a) The patient at first evaluation showed severe wasting and weakness of the shoulder and arm muscles with flail arm–like syndrome. (b) Subtype C PCR. Lane 1: DNA ladder, lane 2: Patient's sample, lane 3: positive control, lane 4: negative control. The 138-bp band represents HIV-1 subtype C, while the 232-bp band represents HIV-1 common band. (c) At 3.5 years' follow-up, the wasting and weakness was stationary on HAART therapy

A 34-year-old unmarried man presented in March 2003 with gradually progressive symmetric wasting and weakness of the proximal muscles of the upper limbs of 2 years' duration. He developed fasciculations over the arms, with rapid worsening of his symptoms over the last 6 months. There was no weakness or wasting of the distal muscles. He gave a history of multiple heterosexual contacts. Examination revealed normal cranial musculature. There was severe wasting of the shoulder girdle and arm muscles, with hypotonia and a power of MRC grade 1-2 [Figure 1a]. The distal muscles in the upper limbs were normal and so were the lower limb muscles. The deep tendon reflexes were absent in the upper limbs and normal in the lower limbs. There was no history of other manifestations of HIV 1 infection. The patient had got his HIV status tested during 2002 as he had had multiple contacts; the test had been reported as positive. He was not started on any treatment.

The detection of anti-HIV antibodies at our institute during 2003 was carried out according to the guidelines from the National AIDS Control Organization (NACO, India). The patient was given pretest and post-test counseling and informed consent was obtained before testing. HIV-1 subtype C–specific polymerase chain reaction (PCR) was carried out on the genomic DNA sample as described earlier by Siddappa *et al*. to investigate whether the HIV infection belonged to HIV-1 clade C.[10] In this subtype–specific PCR, two products were obtained: The 138 bp (HIV-1 clade C–specific product) and another 232 bp product that is common for all HIV-1 subtypes. Hence, this sample harbored the HIV-1 clade C virus. HIV-1 subtype C-specific bands were seen in the PCR [Figure 1b]. The CD_4_ cell count was carried out using a fluorescent-activated cell sort count (FACS count, Becton Dickenson, Singapore). His CD_4+_ T cell, CD_8_, and CD_3_ counts done in May 2003 were 142/ mm^3^, 1438/mm^3^, and 1873/mm^3^, respectively. Cerebrospinal fluid (CSF) analysis showed 2 lymphocytes/mm^3^, glucose level of 42 mg%, and protein of 36 mg%. CSF antitoxoplasma antibodies and cryptococcal antigen were negative. Serum and CSF VDRL were nonreactive. Electromyography (EMG) revealed evidence of chronic denervation, with reinnervation in the arm muscles. EMG of the lower limbs was normal. Motor and sensory nerve conductions were normal, with no evidence of conduction block. Muscle biopsy from the left biceps showed neurogenic atrophy. Neuropsychological assessment was normal.

**Figure 2a and b:**
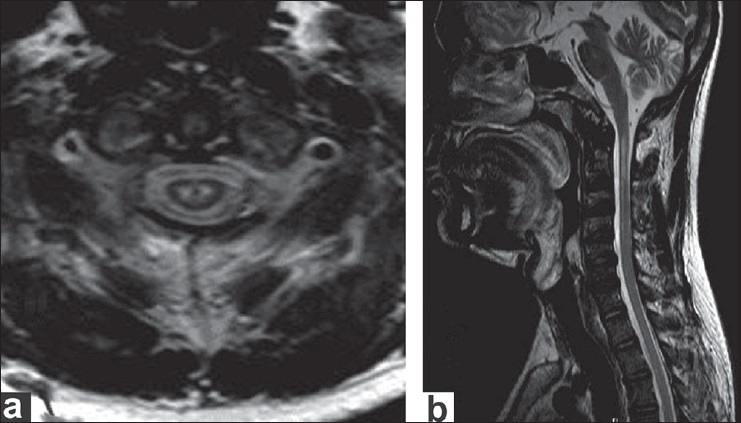
(a) Axial T2-weighted image at C1 vertebral level showing two focal T2 hyperintensities in the central part of spinal cord corresponding to the central grey matter of the cord. (b) Sagittal T2-weighted image showing ill-defined linear hyperintensity of the spinal cord extending between C1 and C4 vertebral body levels

The patient was started on antiretroviral therapy in November 2003 with a combination of lamivudine (150 mg), stavudine (30 mg), and nevirapine (200 mg) and is currently on follow-up. The progression of the wasting and weakness had almost ceased after commencing HAART (highly active antiretroviral therapy) and at the end of 3.5 years follow-up the patient's symptoms continued to be stable; also, he did not develop any other illness related to HIV-1 infection. He was re-evaluated in July 2007. His upper limb proximal muscle weakness and wasting had not progressed [Figure 1c]. His cognitive functions were normal. He underwent multimodal evoked potentials, including visual evoked potentials, brainstem auditory response, and somatosensory evoked potential, all of which were normal. CSF protein and glucose level and cell count were normal. A repeat brachioradialis muscle biopsy showed only neurogenic atrophy. The plasma viral load was undetectable; unfortunately, we had not checked the plasma viral load during 2003 so we cannot comment on this. Serum protein electrophoresis was normal. Chest x-ray showed no abnormal findings. MRI of the brain and spinal cord was done on a 3 Tesla machine. Brain scan showed evidence of diffuse cerebral and cerebellar atrophy. Signal intensity changes in the form of hyperintensity in T2-weighted and FLAIR images and isointensity in T1- weighted images were seen in the white matter of both superior frontal and precentral gyri and periventricular white matter; there was no contrast enhancement. T2- weighted axial slices through the cervical cord revealed symmetrically distributed hyperintense foci in the central part of the spinal cord. A part of these lesions was hypointense on T1-weighted images. [Figure 2a] The sagittal T2-weighted image showed an ill-defined linear hyperintensity in the central part of the spinal cord, extending between C1 and C4 vertebral body levels [Figure 2b]. His repeat CD_4_ and CD_3_ counts were 460/ mm^3^ and 2506/mm^3^, respectively.

This patient continues to take his HAART regimen regularly.

## Discussion

Our young patient of 34 years had features of flail arm syndrome, an ALS-like disorder.[11] The illness had progressed over 2 years and had later attained a stable course with HAART. Other causes for bibrachial amyotrophy, such as inflammatory demyelinating polyneuropathy and multifocal neuropathy with conduction blocks, which can mimic MND, could be excluded as the electrophysiologic features of these disorders were absent. Since 1989, most patients with ALS are investigated for HIV status at our institution and till date we have diagnosed ALS-like syndrome in only two patients, including the present case.[6] About 1330 patients have been diagnosed to have HIV-1 infection associated with neurological manifestations during this period, but only two (0.15%) patients have had features of ALS-like syndrome without other manifestations of HIV-1 infection.

In one study conducted over a 13-year period, six patients with HIV-1 infection in a large series of 1700 patients were confirmed to have an ALS-like disorder, i.e., a frequency of 3.5 per 1000, which greatly exceeds the expected incidence of ALS in the general population of 0.4-1.76 per 100,000.[2] Reports of HIV-1–associated MND strongly suggest that the association between HIV-1 and ALS is not merely coincidental but etiologically related, and an autopsy-confirmed case has clearly shown loss of motor neurons in the cervical and lumbar horns.[12] Furthermore, there are several reports describing stabilization/recovery/improvement of ALS symptoms after starting treatment with HAART.[4,6,14,15] Our patient, who has been receiving HAART since 3.5 years, has also not shown progression in the neurological deficits nor has he developed fresh systemic or neurological symptoms related to HIV-1 infection. This patient is still independent for most activities of daily living and his motor disability has remained the same as it was at the start of HAART. The CD_4_ counts had also improved significantly at the last follow-up.

Although HIV-associated MND may be indistinguishable from classical sporadic ALS, more often it is characterized by a variably progressive lower motor neuron disorder affecting the limbs or the bulbar muscles—a clinical phenotype similar to sporadic progressive muscular atrophy.[1,2,6,11,16,17]

MRI of the brain revealed findings suggestive of HIV- associated cerebral changes. However, there was no evidence of cognitive impairment. The focal, bilateral, hyperintense signals in the cervical spinal cord indicate selective involvement of the central gray matter and this may correlate with the patient's clinical manifestations. Such spinal cord lesions have not been described earlier among HIV-1 infection–associated lower motor neuron syndrome. In one series, six patients with contrast MRI of the brain and the entire spinal cord and roots showed normal findings in all except one patient; this patient had diffuse white matter lesions in the cerebral hemispheres that were highly suggestive of AIDS dementia complex.[2] In the earlier report from India, the patient had diffuse atrophy of brain and a normal spinal cord on MRI.[6] In another report by MacGowan *et al*. contrast MRI of the brain and the cervicothoracic spine was normal except for abnormally enhancing T2 signal in the left brachium pontis and the right lateral medulla.[18] One study reported that somatosensory evoked potential (SSEP) was normal and ruled out any sensory conduction abnormality.[2] SSEP from the upper and lower limbs in our patient were also normal.

The viral theory of MND has been revived over the last one decade, following observations of HIV-1–associated MND;[1,12,13,16,18] another retrovirus, HTLV-1, is also known to cause myelopathy, and the majority of the affected patients have lower motor neuron signs.[19,20] HIV is known to trigger the immune response, with secretion of several cytokines in the CSF, and the toxicity of these has been proposed as a mechanism for neuronal death in ALS.[21] The present case and earlier reports on ALS–like syndrome in HIV-1 infection, particularly lower motor neuron syndromes, emphasizes the need to look for ALS associated with HIV infection. This is important as treatment with HAART may improve/stabilize the neurological deficits.

Our patient did not show improvement in the muscle atrophy and weakness of the affected muscles; rather, there was stabilization in the deficits, which had been steadily progressive prior to commencement of the HAART regimen.
